# Drought tolerance of *Aspergillus violaceofuscus* and *Bacillus licheniformis* and their influence on tomato growth and potassium uptake in mica amended tropical soils under water-limiting conditions

**DOI:** 10.3389/fpls.2023.1114288

**Published:** 2023-03-01

**Authors:** Raji Muthuraja, Thangavelu Muthukumar, Chittamart Natthapol

**Affiliations:** ^1^ Department of Soil Science, Faculty of Agriculture, Kasetsart University, Bangkok, Thailand; ^2^ Department of Botany, Bharathiar University, Coimbatore, India

**Keywords:** microorganisms, polyethylene glycol, tomato growth, mica, enzyme activity

## Abstract

Drought is a significant abiotic stress that alters plant physiology and ultimately affects crop productivity. Among essential plant nutrients, potassium (K) is known to mitigate the deleterious effect of drought on plant growth. If so, K addition or inoculation of potassium solubilizing microorganisms (KSMs) that are tolerant to drought should promote plant growth during water stress. Therefore, in this study, K solubilizing *Aspergillus violaceofuscus* and *Bacillus licheniformis*, isolated from saxicolous environments, were tested for their capacity to tolerate drought using different molecular weights (~4000, 6000, and 8000 Da), and concentrations (0, 250, 500, 750, 1000, and 1250 mg/L) of polyethylene glycol (PEG) under *in vitro* conditions. The results showed that high concentrations (750 and 1000 mg/L) of PEG with different molecular weight considerably improved bacterial cell numbers/fungal biomass and catalase (CAT) and proline activities. Moreover, the ability of KSMs alone or in combination to impart drought tolerance and promote plant growth in the presence and absence of mica (9.3% K_2_O) supplementation was tested in Alfisol and Vertisol soil types under greenhouse conditions. The results revealed that the tomato plants inoculated with KSMs individually or dually with/without mica improved the physiological and morphological traits of the tomato plants under drought. Generally, tomato plants co-inoculated with KSMs and supplemented with mica were taller (2.62 and 3.38-fold) and had more leaf area (2.03 and 1.98-fold), total root length (3.26 and 8.86-fold), shoot biomass (3.87 and 3.93-fold), root biomass (9.00 and 7.24-fold), shoot K content (3.08 and 3.62-fold), root K content (3.39 and 2.03-fold), relative water content (1.51 and 1.27-fold), CAT activity (2.11 and 2.14-fold), proline content (3.41 and 3.28-fold), and total chlorophyll content (1.81 and 1.90-fold), in unsterilized Alfisol and Vertisol soil types, respectively, than uninoculated ones. Dual inoculation of the KSMs along with mica amendment, also improved the endorrhizal symbiosis of tomato plants more than their individual inoculation or application in both soil types. These findings imply that the *A. violaceofuscus* and *B. licheniformis* isolates are promising as novel bioinoculants for improving crop growth in water-stressed and rainfed areas of the tropics in the future.

## 1 Introduction

Drought is a major abiotic factor that decreases agricultural performance owing to negative impacts on plant growth and productivity (Slama et al., 2015; Kaya et al., 2020; Kosar et al., 2020). Estimates indicate a 17-70% reduction in crop yields due to different types of stress, and the majority of this yield loss is due to drought (Hubbard et al., 2012; Ahmed et al., 2019; 2021). The response to drought tolerance is a complex trait that depends on several factors, including the environment, genotype, development stage, and stress severity and duration (Fahad et al., 2017; Sallam et al., 2019). Potassium (K) availability in the soils is limited under drought conditions (Bahrami-Rad and Hajiboland, 2017). Nevertheless, increased availability of K under water-limiting conditions mitigates drought stress characteristics, including the reduction in growth traits, plant water status, chlorophyll content, and a decline in the relative water content (RWC) (Munsif et al., 2022). Plants have various physiological and defensive mechanisms to alleviate water stress damage under increased K nutrition, including changes in physiological and morphological characteristics (Gurumurthy et al., 2019) like the alterations in proportional biomass allocation between roots and shoots under water-limiting conditions (Chandra et al., 2019; Yu et al., 2020). Under drought conditions, stomatal closure can limit water loss through transpiration and photosynthesis (Fan et al., 2020). Furthermore, plants under water deficit can enhance the intervention of reactive oxygen species (ROS) through the modulation of antioxidant defenses and enzymes intended to reduce oxidative damage caused by ROS (Uzilday et al., 2012; Kasote et al., 2015; Kaya et al., 2020; Kosar et al., 2020). Increased availability of K in the soil can upregulate the antioxidant and osmolyte protective mechanisms in plants under stress. Consequently, plants with greater antioxidant capacity are more resilient to soil dryness. Earlier studies have shown that in addition to the antioxidant enzymatic system, proline activity is also a key component of plant tolerance to drought and serves as a defensive mechanism (Liang et al., 2013; Laxa et al., 2019) under drought conditions.

Mining of K through crop production has rendered agricultural soils K deficient, and synthetic K fertilizers are often used to replenish K in cultivated soils (Basak, 2019). In addition to synthetic K fertilizers, silicate mineral deposits like mica containing K are often used for fertilizing crops in the tropics. Mica is often used as the insoluble K source to determine the K solubilizing ability of microorganisms under *in vitro* conditions (Muthuraja and Muthukumar, 2021a; Muthuraja and Muthukumar, 2021b). However, mica containing 6–10% K_2_O is used for fertilizing field crops due to its slow mineralization and extended K availability to crop plants (Pramanik and Kalita, 2019).

Currently, loss in soil fertility due to the irrational use of chemical fertilizers and increased incidence of abiotic stresses such as drought due to climate change are major challenges threatening crop production and food security worldwide (Waqas et al., 2019; Fu et al., 2022). It is, therefore, crucial to develop strategies to improve crop growth under water-limiting conditions. One of the ways to enhance the growth and yield of crops in drought-affected agricultural soils is to effectively utilize the potential plant growth-promoting microorganisms (PGPM) in food production (Gowtham et al., 2020; Mishra et al., 2020). Beneficial microorganisms associated with the rhizosphere, phyllosphere, and endosphere of plants that influence plant health and nutrient conditions may also face many environmental stresses, especially drought (Dong et al., 2019). These PGPMs also vary in their tolerance to water-limiting environments, as revealed by *in vitro* studies (Turco et al., 2005; Ghosh et al., 2019). Polyethylene glycol (PEG) is a petroleum-derived polyether compound that is often used to induce moisture stress under *in vitro* conditions (Ghosh et al., 2019; Mishra et al., 2021).

Previously, inoculation of PGPMs, such as *Arthrobacter*, *Azotobacter*, *Bacillus*, *Azospirillum*, *Enterobacter*, *Burkholderia*, *Pseudomonas*, *Paenibacillus*, *Aspergillus*, and *Trichoderma* has been shown to improve drought-tolerance and promote the growth and development of different crop plant taxa under water-stressed conditions (Alwhibi et al., 2017; Chandra et al., 2019; Ismail et al., 2019; Gowtham et al., 2020; Ismail et al., 2020; Akhtar et al., 2021; Yasmin et al., 2021; Zhao et al., 2023). Nonetheless, studies on the impact of using specific microbes alone or in combination with natural inorganic fertilizers on crop development under drought conditions are limited (e.g., Bargaz et al., 2018). Tomatoes (*Solanum lycopersicum* L.) are important horticultural crops susceptible to drought stress during their development (Gowtham et al., 2020). In addition, tomatoes are often used as a model organism for understanding various applied and fundamental aspects of plant research (Anwar et al., 2019). Moreover, tomatoes are also sensitive to heat and water stress, which affect their growth (Cui et al., 2020). The catalase (CAT) activity and proline accumulation help plants to surpass the oxidative damage induced by drought stress (Laxa et al., 2019). Recently, more attention has been devoted to cognizing the antioxidant defense pathway in plants growing under drought (Hasanuzzaman et al., 2018; Hussain et al., 2018). In this aspect, potassium solubilizing microorganisms (KSMs) are significant PGPMs that help increase drought resistance while enhancing secondary metabolite content (Cappellari et al., 2013; Singh et al., 2016).

A diverse range of natural endophytic fungi naturally colonizes plant roots, with arbuscular mycorrhizal (AM) fungi belonging to the subphylum Glomeromycotina and phylum Mucoromycota being among the most common root colonizers (Schüβler et al., 2001; Field et al., 2016; Spatafora et al., 2016). Similarly, the dark septate endophyte (DSE) fungi belonging to Ascomycota also colonize plant roots in natural and agroecosystems. These fungi form symbiotic relationships with crop plants (Smith et al., 2011; Spatafora et al., 2016), and there is evidence that AM fungi can improve host plant tolerance to drought stress (Begum et al., 2019; Begum et al., 2021). In general, AM symbiosis alleviates the stress caused by drought in plants by improving the direct absorption of water, and nutrients, increasing the osmotic and antioxidant activity (Begum et al., 2019; Begum et al., 2021). Nevertheless, the impact of KSM inoculation on native AM and DSE fungal symbiosis is not well resolved.

Saxicolous (rock) microbial isolates have not been thoroughly studied for their role in improving the nutrient content of plants grown in different soil types under drought (Muthuraja and Muthukumar, 2021a). Further, the tolerance of KSMs like *Aspergillus violaceofuscus* and *Bacillus licheniformis* previously isolated from saxicolous habitats to drought stress is also unknown (Muthuraja and Muthukumar, 2022). Therefore, the present study aims to (i) assess the efficacy of *A. violaceofuscus* and *B. licheniformis* to tolerate drought stress induced by PEG of different molecular weights and concentrations under *in vitro* conditions; (ii) determine the influence of carrier-based formulation of the KSM isolates individually with/without mica supplement on tomato growth, chlorophyll content, and plant K content under drought conditions in different soil types, and (iii) explore the effect of co-inoculation of the above KSMs on the growth, RWC, antioxidant enzyme activities; and AM and DSE fungal root colonization in tomato plants under drought stress.

## 2 Materials and methods

### 2.1 Biological materials

Both *in vitro* and *in vivo* experiments were conducted using previously characterized fungi (*A. violaceofuscus*-MH220545) and bacteria (*B. licheniformis*-MN718157) from saxicolous habitats (Muthuraja and Muthukumar, 2021a; Muthuraja and Muthukumar, 2021b; Muthuraja and Muthukumar, 2022).

### 2.2 Drought tolerance of KSMs

The drought tolerance of *B. licheniformis* and *A. violaceofuscus* was assessed based on the bacterial cell numbers and hyphal mass production in different concentrations (0, 250, 500, 750, 1000, and 1250 mg/L) and molecular weights (4000, 6000, and 8000) of PEG in lysogeny broth (LB) (Hu et al., 2007; Omara and Elbagory, 2018). The experiment without PEG served as a control. The solid or broth medium was supplemented with different concentrations of PEG and solidified using the gelling agent phytagel (Klimaszewska et al., 2000). To examine the drought tolerance of the bacterium, the sterilized flasks containing 50 mL of LB medium spiked with varying concentrations of PEG were inoculated with 3×10^6^ CFU/mL of freshly prepared bacterial culture that was incubated at 37°C in a shaking incubator (120 rpm) (Hu et al., 2007). Further, to assess the drought tolerance of *A. violaceofuscus*, each conical flask containing potato dextrose broth supplemented with different concentrations of PEG was inoculated with a 10 mm mycelial disk and incubated at 28°C. The experiment consisted of three replications for each concentration and different molecular weights of PEG. The experimental approach described in our previous study (Muthuraja and Muthukumar, 2022) was used for culture maintenance and microbial growth quantification. The proline content in KSM cultures was determined according to Kahraman and Karaderi (2021).

### 2.3 *In vivo* study

#### 2.3.1 Preparation of inoculum

The rice husk-based inoculum was prepared following the procedure mentioned in our earlier work (Muthuraja and Muthukumar, 2022). The rice husk used for the inoculum preparation was purchased from a local market. A bacterial suspension containing 3×10^6^ CFU/mL (120 mL of log-phase growing culture) was inoculated into the sterilized carrier and cured for a week (Muthuraja and Muthukumar, 2022). Then, under aseptic conditions, 10 mm discs of the potato dextrose agar (PDA) subculture fungus were inoculated into the carrier and carefully sealed.

#### 2.3.2 Greenhouse experiment

The potential of KSMs in improving plant growth and K content under drought stress was examined according to the methods described by Muthuraja and Muthukumar (2022). In addition, around 2 g of the carrier-based bacterial (67.65 ± 1.46 cfu/g) and fungal (57.00 ± 1.49 cfu/g) inoculum was introduced to the respective treatments.

#### 2.3.3 Experimental design

The 2 × 2 × 2 × 3 factorial experiments consisted of four factors, namely two soil types (Alfisol and Vertisol), two soil conditions (sterilized and unsterilized), two levels of mica amendment (amended and unamended), and three levels of microbial inoculation (*A. violaceofuscus* and *B. licheniformis* inoculated individually or in combination). Tomato seedlings were prepared in the greenhouse by sowing presoaked seeds in seedling trays containing heat sterilized (121 °C at 15 psi) soil - sand mixture (1:3, v:v). When a pair of true leaves (7 days after sowing) developed, two seedlings were taken and planted into each black polybag (19 cm × 13 cm, height × width) containing 0.30 kg of unsterile or sterile soil as per the treatments (Muthuraja and Muthukumar, 2022). About 30 g of mica was added to the soil and thoroughly mixed for treatments involving mica. In addition, around 2 g of the carrier-based bacterial (67.65 ± 1.46 × 10^5^ cfu/g) and fungal (57.00 ± 1.49 × 10^3^ cfu/g) inoculum was placed below the seedlings in the respective treatments. For treatments involving both the KSMs a 2 g mixture of the two inoculums (in equal proportion) was inoculated. Treatments not involving KSMs were mock-inoculated with a similar quantity of heat-sterilized KSM inoculums (Muthuraja and Muthukumar, 2022).

After one week, adequate irrigation was provided initially to all the plants irrespective of treatments at regular intervals (once a day). The seedlings were maintained in this condition for the first 20 days of their establishment and development. After 20 days, the watering frequency (100 mL/bag) was reduced to twice a week. Initially, plants struggled to adapt to the reduced irrigation frequency but adapted within 3–4 cycles to the available moisture conditions. The last five cycles of watering were done after the plants exhibited wilting. Finally, sterile deionized water was applied to non-stressed plants. To assess changes in pH and available K, about 100 g of soil was collected from each replicate of each treatment at the end of the study.

#### 2.3.4 Assessment of plant growth and tissue nutrients

The plant roots were washed with tap water to free off adhering dirt. The shoot length and leaf area were determined by the procedure developed by Giday et al. (2013). The total root length was calculated using modified Newman’s line intersect technique (Tennant, 1975). After drying for 48 h in a preheated oven at 60–65°C, the biomass of the shoots and roots was assessed. Half the freshly harvested shoots and roots from each treatment were kept at -20°C for biochemical testing. The tissue K content of plants under drought was investigated using the methods described by Muthuraja and Muthukumar (2022).

#### 2.3.5 Percentage relative water content

Fresh leaves from each sample were weighed immediately (Fresh weight - FW) after the plant was harvested to evaluate the RWC. After that, the leaves were immersed in distilled water for 4 h before being examined for the turgid weight (TW). The leaves are then dried in a preheated oven at 70°C for 24 h and weighed by dry weight (DW). Sharp et al. (1990) proposed the technique for estimating the percentage of RWC as follows.


%RWC=(FW−DW)/(TW–DW)×100

#### 2.3.6 Chlorophyll content estimation

The methods proposed by Parry et al. (2014) were used to determine the total chlorophyll content of the leaves. Fresh leaf samples (100 mg) were homogenized using a mortar and pestle in 10 mL of 80% acetone. For 10 minutes, the homogenate was centrifuged at 4000×g. The supernatant was utilized to calculate the chlorophyll concentration. The absorbance of the samples was measured at 645 and 663 nm using a spectrophotometer (Shimadzu, Japan).

#### 2.3.7 Measurement of antioxidant and non-antioxidant enzyme activity

After the end of the experimental period, the enzymatic (CAT) and non-enzymatic (proline) analysis of bacterial cells, fungal biomass (*in vitro*), and plant samples (*in vivo*) were determined according to the method described by Muthuraja and Muthukumar (2022).

#### 2.3.8 Evaluation of endophytic fungal root colonization

The roots cut into 1 cm pieces were cleared with 3% KOH at 85°C for 45 min, acidified with 5 N HCl, and stained with trypan blue (0.05%) for 24 h (Koske and Gemma, 1989). To make the root squashes, the individual dyed root bits were placed on glass slides with clear lactoglycerol, sealed with cover glasses, and squeezed. We used an Olympus BX51 compound microscope to examine the slides. For each root specimen, 150 intersections were observed at 40×. The percentage (%) of root length with total AM and DSE fungal colonization and fungal structures was assessed according to McGonigle et al. (1990). Microscopic images were captured with a ProgRes 3 digital camera attached to an Olympus BX51 trinocular microscope.

### 2.4 Statistical analysis

The Kolmogorov-Smirnov test was used to evaluate the data for normality, and all measured values were expressed as mean ± standard errors (SE) (Storer et al., 2017). The data failing to satisfy normality were log-transformed prior to statistical analysis. Multiple comparisons were made using analysis of variance (ANOVA), and when the F values were significant (p< 0.05), Duncan’s multiple range test (DMRT) was employed to compare means. Finally, Pearson’s correlation was used to examine the correlations between the variables chosen. The IBM SPSS program version 21.0 was used for all statistical analyses. GraphPad Prism 6.0 and OriginPro 2021 software were used to compile the data and create the graphical images.

## 3 Results

### 3.1 Drought tolerance of *A. violaceofuscus* and *B. licheniformis*

**Figure 1:**
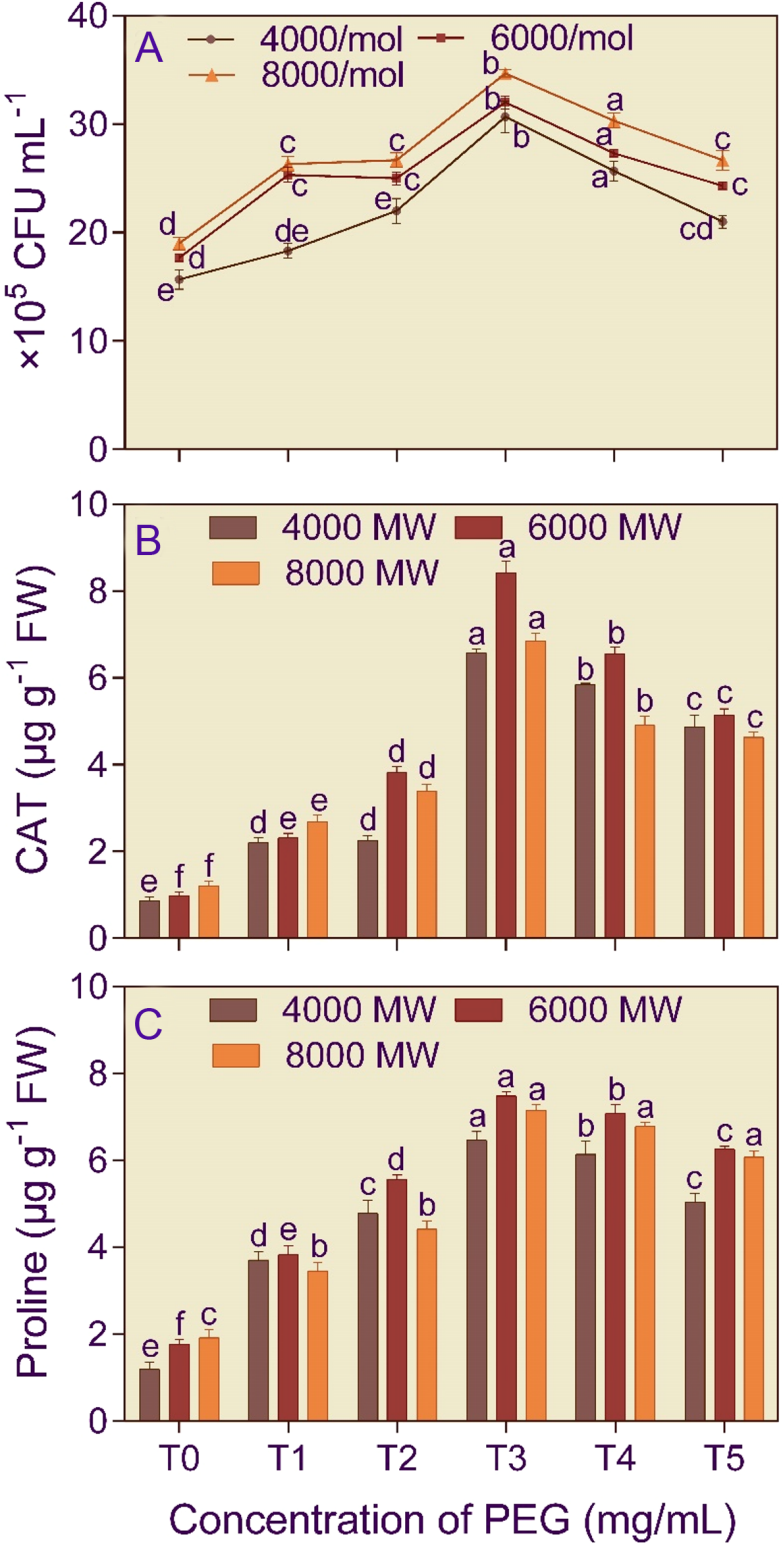
Growth **(A)**, catalase activity **(B)**, and proline content **(C)** of *Bacillus licheniformis* (KSB) in the presence of different molecular weights (4000 MW, 6000 MW, and 8000 MW) and concentrations (T0, 0 mg/L; T1, 250 mg/L; T2, 500 mg/L, T3, 750 mg/L; T4, 1000 mg/L; and T5, 1250 mg/L) of polyethylene glycol (PEG). Error bars represent ± 1 standard error. Bars for a molecular weight of PEG bearing the same letter(s) are not significantly (p > 0.05) different according to Duncan’s Multiple Range Test.

The cell number of *B. licheniformis* was 1.81–1.96 higher at 750 mg/L PEG amendment compared to the control (**Figure 1A**). The PEG concentration (F_5,53_ = 137.145) and PEG molecular weight (F_2,53_ = 71.602) significantly (p< 0.001) affected bacterial growth. Furthermore, the two-way interaction, concentration × PEG molecular weight (F_10,53_ = 2.343) significantly (p< 0.05), affected the bacterial growth.

**Figure 2:**
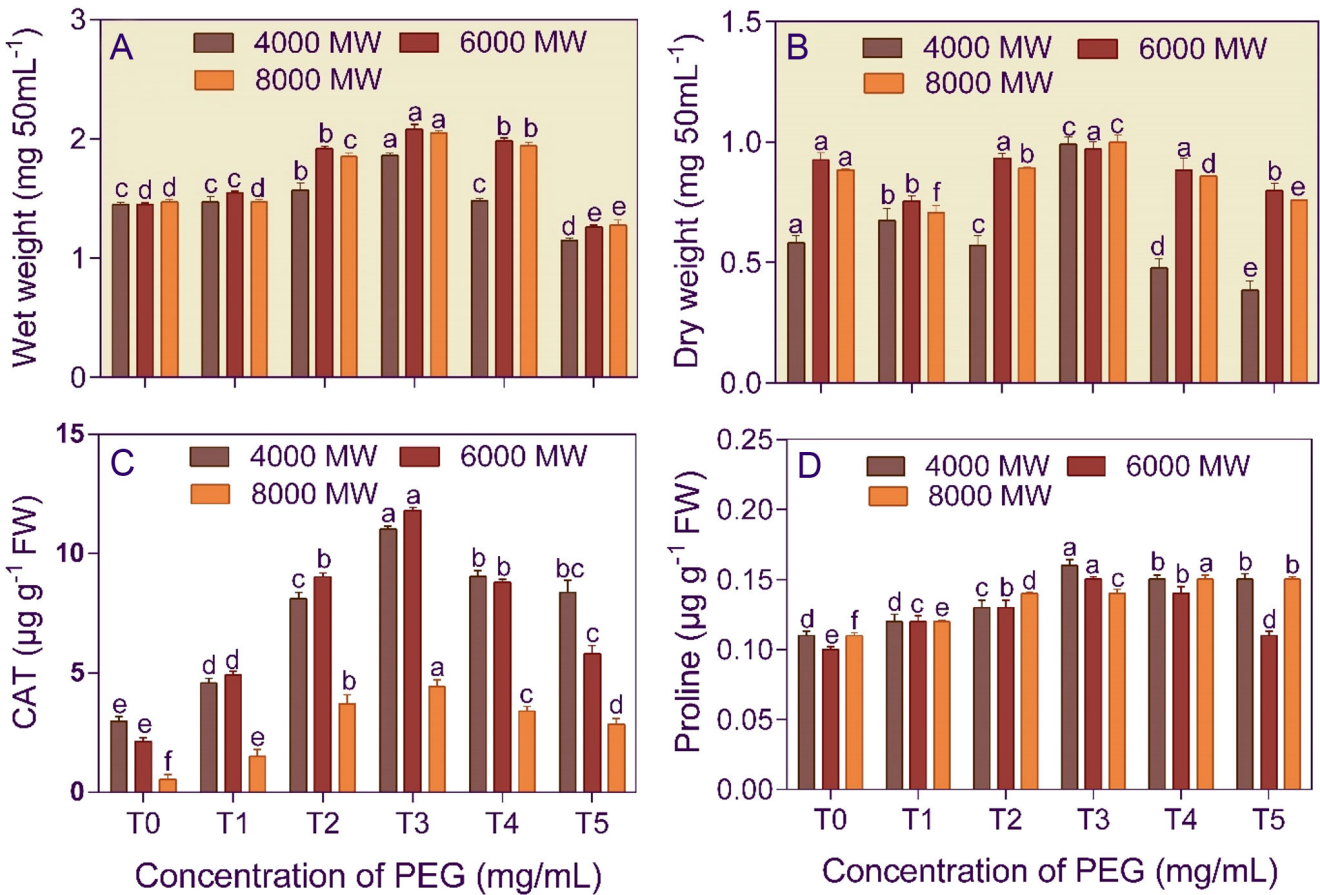
Growth **(A, B)**, catalase activity **(C)**, and proline content **(D)** of *Aspergillus violaceofuscus* (KSF) cultured in the presence of different molecular weights (4000 MW, 6000 MW, and 8000 MW) and concentrations (T0, 0 mg/L; T1, 250 mg/L; T2, 500 mg/L, T3, 750 mg/L; T4, 1000 mg/L; and T5, 1250 mg/L) of polyethylene glycol (PEG). Error bars represent ± 1 standard error. Bars for a molecular weight of PEG bearing the same letter(s) are not significantly (p > 0.05) different according to Duncan’s Multiple Range Test.

Amendment of the culture media with 750 mg/L PEG increased the fresh and dry mass of *A. violaceofuscus* by 1.28, 1.43, and 1.40-fold and 1.41, 1.42, and 1.41-fold, respectively, than control (**Figures 2A, B**). Furthermore, the PEG concentration (F_5,53_ = 396.504 and 778.725) and PEG molecular weights (F_2,53_ = 130.784 and 461.635) significantly (p< 0.001) affected the fungal wet and dry mass. Moreover, the two-way interaction, concentration × PEG molecular weight (F_10,53_ = 18.293 and 84.291), was significant (p< 0.001) for the wet and dry fungal mass.

### 3.2 Catalase activity in KSMs under drought

The bacterial isolate *B. licheniformis* (5.66–8.55-fold increase) and the fungal isolate *A. violaceofuscus* (3.68 – 8.10-fold increase) had the highest CAT activity at T3 concentration in different molecular weights of PEG, compared to T0 treatment (**Figures 1B**, **2C**). The PEG concentration (F_5,53_ = 139.83 and 661.586) and PEG molecular weight (F_2,53_ = 84.241 and 132.230) significantly (p< 0.001) affected the CAT activity of *B. licheniformis* and *A. violaceofuscus*. Furthermore, the two-way interaction, concentration × PEG molecular weight (F_10,53_ = 26.280 and 47.140), of the KSMs was significant (p< 0.001) for CAT activity.

### 3.3 Proline content in KSMs under PEG

The proline content of *B. licheniformis* culture was 3.34–5.43-fold higher than the control when the medium was supplemented with 750 mg/L of different molecular weight PEG (**Figure 1C**). In the case of *A. violaceofuscus*, the proline content was 1.46 and 1.49-folds higher at 750 mg/L n in P_4000_ and P_8000_, respectively, than in control, while in P_8000_ it was 1.38-fold higher in 1000 mg/L compared to control (**Figure 2D**). The PEG concentration (F_5,53_ = 224.588 and 146.400) and PEG molecular weight (F_2,53_ = 16.366 and 40.875) significantly (p< 0.001) influenced the proline content of *B. licheniformis* and *A. violaceofuscus*. Also, the two-way interaction, concentration × PEG molecular weight, was significant for *B. licheniformis* (F_10,53_ = 1.996; p< 0.05) and *A. violaceofuscus* (F_10,53_ = 17.175, p< 0.001).

### 3.4 Greenhouse experiment

#### 3.4.1 Effect of KSMs on plant growth

The KSM inoculation, along with mica amendment, soil types, and conditions, positively (p < 0.001) influenced the shoot and total root lengths, leaf area, shoot and root dry masses (**Table 1**). However, soil conditions failed to influence (p >0.05) the root/shoot (R/S) ratio of the tomato plants (**Table 1**). All the two-way interactions among the factors were significant for all the variables except for the interactions soil condition × soil type, mica amendment × soil type, and mica amendment × soil condition for R/S ratios. Similarly, all the three-way interactions were significant for all the variables except for the interactions mica amendment × soil type × soil condition for the shoot and total root lengths, mica amendment × microbial inoculation × soil type interaction for the dry shoot and root weights, and mica amendment × microbial inoculation × soil condition interaction for shoot dry weight. The four-way interaction among the factors was significant for all the studied variables except leaf area and R/S ratios (**Table 1**).

**Table 1:** Results for MULTI-ANOVA analysis for the effect of potassium solubilizing microorganisms inoculation, mica amendment, soil type, and soil condition on plant growth, potassium uptake, and physiological and biochemical variables in tomato under drought.

^†^Source	df	^††^Plant growth parameters and enzyme activity											
		SE	LA	TRE	SDM	RDM	SKC	RKC	R/S ratio	RWC	TCH	CAT	PRO
M	1,95	5.869*	9.745**	32.762***	186.513***	307.252***	93.664***	178.341***	7.615**	22.213***	738.922***	12.753***	1.315ns
I	3,95	2125.3***	564.754***	1590.3***	534.720***	722.045***	938.914***	807.655***	46.072***	54.041***	993.090***	3353.289***	3208.100***
ST	1,95	181.848***	2.886*	705.787***	156.055***	33.697***	396.805***	512.069***	30.215***	10.884**	1.158ns	321.528***	1316.138***
SC	1,95	959.081***	297.888***	1915.3***	262.442***	252.117***	640.715***	591.828***	0.531ns	4.939*	231.614***	116.402***	474.862***
M * I	3,95	19.250***	4.046*	66.495***	21.453***	43.148***	33.421***	31.879***	4.588**	0.574ns	30.874***	49.167***	451.363***
M* ST	1,95	7.211**	0.017ns	30.786***	15.607***	10.157**	6.141*	15.429***	0.597ns	0.149ns	46.065***	43.985***	164.695***
M *SC	1,95	7.689**	19.101***	78.457***	0.530ns	3.968*	19.395***	1.718ns	0.000ns	0.169ns	24.127***	1.328ns	8.187**
I * ST	3,95	37.789***	6.907***	45.308***	9.759***	2.477*	47.714***	22.751***	4.576**	0.929ns	6.139**	16.841***	150.412***
I * SC	3,95	7.274***	7.356***	43.275***	6.147***	22.082***	12.415***	38.412***	2.445*	0.204ns	27.255***	11.159***	25.835***
ST * SC	1,95	2.866*	8.448**	103.032***	8.423**	8.424**	79.123***	0.046ns	0.468ns	1.605ns	67.502***	6.898***	59.886***
M * I * ST	3,95	8.914***	3.796*	16.292***	2.031ns	1.285ns	42.000***	5.770***	1.136ns	1.646ns	1.525ns	19.603***	267.274***
M * I * SC	3,95	18.063***	3.880*	30.341***	1.646ns	2.592*	14.280***	10.328***	0.730ns	1.447ns	6.274**	6.574***	14.983***
M * ST * SC	1,95	0.767ns	6.659*	0.098ns	15.915***	5.989*	4.107*	77.706***	29.252***	13.299***	1.522ns	1.160ns	10.390***
I * ST *SC	3,95	49.616***	1.154ns	11.523***	6.274***	11.804***	9.564***	5.046**	2.190*	1.517ns	9.467**	12.833***	1.889ns
M * I * ST * SC	3,95	10.982***	1.425ns	4.613**	2.549*	3.853*	12.415***	38.992***	0.996ns	0.631ns	5.097**	9.069***	6.636**

^†^M, mica; I, inoculation; ST, soil type; SC, soil condition.

^††^ SE, shoot elongation; LA, leaf area; TRE, total root elongation; SDW, shoot dry mass; RDW, root dry mass; SKC, shoot potassium content; RKC, root potassium content; R/S, root/shoot ratio; RWC, relative water content; THC, total chlorophyll content; CAT, catalase activity; PRO, proline content.

*, **, *** significant at p< 0.05, p< 0.01, p< 0.001 respectively; ns, not significant.

##### 3.4.1.1 Shoot length

The shoot length of KSM dual inoculated tomato plants was 4.10 and 4.22-fold higher in sterile Alfisol soil when compared to negative (without mica) and positive (with mica) control, respectively (**Table 2**). Similarly, the shoot lengths of dual inoculated tomato plants in unsterile Alfisol soil were respectively 2.01 and 2.12-fold higher than the positive and negative control. In Vertisol soil, the shoot length of dual inoculated plants compared to negative and positive control was 2.16 and 2.45-fold and 2.27 and 3.38-fold higher in sterile and unsterile conditions.

**Table 2:** Influence of potassium solubilizing microorganisms (KSMs) inoculation on tomato growth under drought conditions in different soil type and condition.

Treatment	^†^Plant growth parameters							
	Shoot length (cm/plant)		Leaf area (cm^2^/plant)		Total root length (cm/plant)		R/S ratio	
	^*^ST	^*^US	ST	US	ST	US	ST	US
Alfisol								
Without mica								
Control	5.33 ± 0.09^e^	11.90 ± 0.35^d^	5.64 ± 0.04^c^	11.03 ± 0.42^d^	60.15 ± 2.73^f^	254.82 ± 6.00^f^	0.23 ± 0.02^d^	0.26 ± 0.03^bc^
*Bacillus licheniformis*	20.73 ± 0.33^b^	22.70 ± 0.55^b^	15.37 ± 0.52^b^	17.26 ± 1.24^c^	284.17 ± 15.41^e^	434.06 ± 7.86^e^	0.39 ± 0.02^ab^	0.35 ± 0.04^ab^
*Aspergillus violaceofuscus*	15.63 ± 0.26^d^	20.67 ± 0.90^c^	15.60 ± 0.67^b^	21.04 ± 0.78^b^	399.47 ± 6.48^c^	608.37 ± 33.28^d^	0.28 ± 0.03^cd^	0.36 ± 0.01^a^
*B. licheniformis* + *A. violaceofuscus*	21.87 ± 0.38^a^	23.93 ± 0.23^ab^	16.48 ± 0.71^b^	21.59 ± 1.04^ab^	544.17 ± 6.38^a^	822.63 ± 16.93^a^	0.30 ± 0.01^bcd^	0.37 ± 0.05^a^
With mica								
Control	5.20 ± 0.23^e^	9.37 ± 0.09^e^	6.17 ± 0.15^c^	11.58 ± 0.27^d^	40.82 ± 2.50^f^	236.80 ± 13.86^f^	0.23 ± 0.01^d^	0.17 ± 0.02^c^
*B. licheniformis*	15.70 ± 0.29^d^	23.40 ± 0.61^ab^	15.69 ± 0.74^b^	18.43 ± 0.42^c^	282.09 ± 12.34^e^	676.15 ± 11.53^c^	0.46 ± 0.07^a^	0.34 ± 0.02^ab^
*A. violaceofuscus*	16.73 ± 0.12^c^	20.10 ± 0.47^c^	16.92 ± 0.39^ab^	18.62 ± 0.20^c^	316.54 ± 10.96^d^	608.00 ± 13.70^d^	0.48 ± 0.03^a^	0.37 ± 0.04^a^
*B. licheniformis* + *A. violaceofuscus*	21.93 ± 0.33^a^	24.50 ± 0.25^a^	18.40 ± 0.30^a^	23.48 ± 0.64^a^	481.25 ± 13.15^b^	772.71 ± 11.41^b^	0.36 ± 0.01^bc^	0.39 ± 0.02^a^
Vertisol								
Without mica								
Control	10.30 ± 0.49^c^	12.43 ± 0.29^c^	8.27 ± 0.16^e^	12.79 ± 0.77^c^	59.81 ± 3.34^e^	117.15 ± 4.18^f^	0.23 ± 0.03^cd^	0.18 ± 0.01^f^
*B. licheniformis*	17.80 ± 0.30^b^	21.77 ± 0.29^d^	14.03 ± 0.43^d^	17.25 ± 0.36^b^	214.83 ± 8.88^d^	265.26 ± 15.86^e^	0.30 ± 0.03^ab^	0.26 ± 0.01^cde^
*A. violaceofuscus*	18.97 ± 0.29^b^	23.47 ± 0.55^c^	14.39 ± 0.70^d^	19.00 ± 0.43^b^	324.93 ± 25.28^b^	455.50 ± 10.03^d^	0.32 ± 0.02^ab^	0.25 ± 0.02^dc^
*B. licheniformis* + *A. violaceofuscus*	22.20 ± 0.35^a^	28.17± 0.33^b^	18.14 ± 0.52^b^	23.11 ± 0.54^a^	274.15 ± 9.90^c^	501.88 ± 11.87^bc^	0.35 ± 0.01^a^	0.29 ± 0.02^bcd^
With mica								
Control	9.13 ± 0.32^c^	9.60 ± 0.26^f^	7.36 ± 0.55^e^	11.15 ± 0.61^d^	41.48 ± 1.30^e^	68.56 ± 4.41^g^	0.19 ± 0.01^d^	0.21 ± 0.02^ef^
*B. licheniformis*	17.77 ± 0.37^b^	22.57 ± 0.52^cd^	16.26 ± 0.69^c^	17.26 ± 0.53^b^	285.93 ± 3.86^c^	493.68 ± 9.55^cd^	0.26 ± 0.03^bc^	0.32 ± 0.03^bc^
*A. violaceofuscus*	18.03 ± 0.27^b^	23.50 ± 0.58^c^	17.74 ± 0.13^bc^	18.38 ± 0.52^b^	222.30 ± 7.03^d^	538.70 ± 9.52^b^	0.29 ± 0.02^ab^	0.35 ± 0.01^ab^
*B. licheniformis* + *A. violaceofuscus*	22.40 ± 0.61^a^	32.40 ± 0.44^a^	21.55 ± 0.55^a^	22.14 ± 0.49^a^	374.74 ± 10.03^a^	607.34 ± 30.21^a^	0.33 ± 0.03^ab^	0.39 ± 0.01^a^

^†^R/S ratio, root/shoot ratio. ^*^Soil condition: ST, sterile; US, unsterile. Means± standard error in a column for a soil type followed by the same superscript(s) is not significantly (p > 0.05) different according to Duncan’s Multiple Range Test.

##### 3.4.1.2 Leaf area

The leaf area of dual inoculated tomato plants was 2.92 and 2.98-fold larger in sterile Alfisol soil when compared to the negative and positive control, respectively (**Table 2**). Likewise, the leaf area of dual inoculated tomato plants in unsterile Alfisol soil was respectively 1.96 and 2.03-fold larger than the negative and positive control. The leaf area of dual inoculated tomato plants compared to negative and positive control was 2.19 and 2.93-fold and 1.81 and 1.98-fold higher in sterile and unsterile Vertisol soils, respectively.

##### 3.4.1.3 Total root length

The roots of dual-inoculated tomato plants were 9.05 and 11.79-fold longer in sterile Alfisol soil than the roots of tomato plants in the negative and positive control, respectively (**Table 2**). Similarly, the total root lengths of dual inoculated tomato plants in unsterile Alfisol soil were 3.23 and 3.26-fold longer than the positive and negative control. In sterile Vertisol soil, the total root lengths of *B. licheniformis* and dual inoculated plants were 5.34 and 9.03-fold longer than the negative control and positive controls, respectively. However, in unsterile Vertisol soil, the total root lengths of dual inoculated tomato plants were 4.28 and 8.86-fold longer than the negative and positive control, respectively (**Table 2**).

##### 3.4.1.4 Shoot dry weight

**Figure 3:**
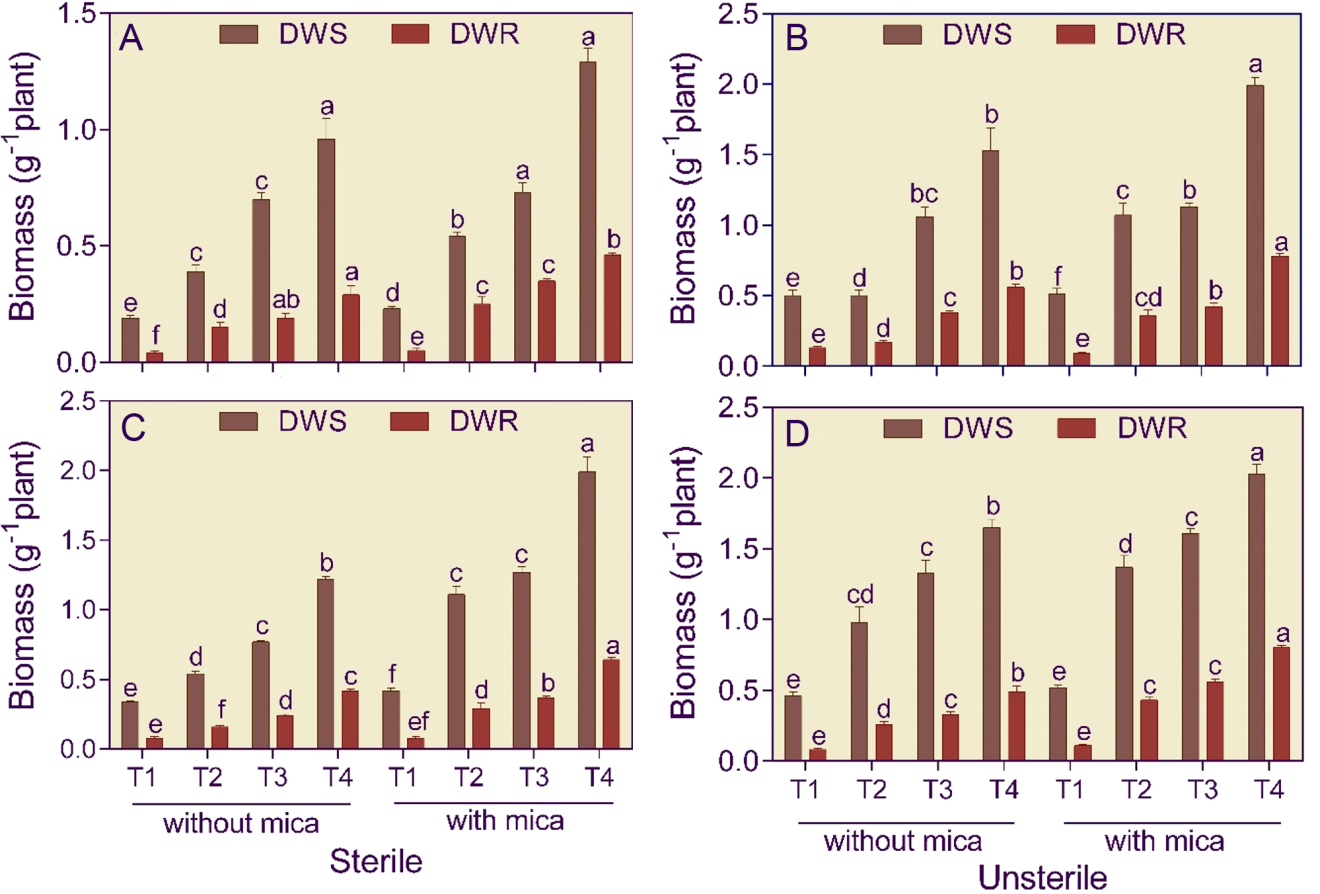
Shoot (DWS) and root (DWR) dry weight of tomato grown in sterile and unsterile Alfisol **(A, B)** and Vertisol **(C, D)** soils amended or unamended with mica and inoculated or uninoculated with potassium solubilizing microorganisms under drought. T1, control (negative and positive); T2, *Bacillus licheniformis* inoculation; T3, *Aspergillus violaceofuscus* inoculation; T4, *A. violaceofuscus* + *B. licheniformis* inoculation. Bars bearing the same alphabet for a variable are not significantly (p > 0.05) different according to Duncan’s Multiple Range Test.

In Alfisol soil, the shoots of dual inoculated tomato plants compared to negative and positive control were 5.05 and 5.62-fold and 3.04 and 3.87-fold heavier in sterile and unsterile conditions, respectively (**Figures 3A, B**). In Vertisol soil, the shoot biomass of dual inoculated tomato plants compared to negative and positive control was 3.55 and 4.70-fold higher in sterile conditions and 3.61 and 3.93-fold higher in unsterile conditions (**Figures 3C, D**).

##### 3.4.1.5 Root dry weight

Roots of dual inoculated tomato plants in Alfisol soil compared to negative and positive control were 6.69 and 5.32-fold and 4.39 and 9.00-fold heavier in sterile and unsterile conditions, respectively (**Figures 3A, B**). In Vertisol soil, the roots of dual inoculated tomato plants compared to negative and positive control were heavier by 5.29 and 8.04-fold in sterile condition and 5.84 and 7.27-fold heavier in unsterile condition (**Figures 3C, D**).

##### 3.4.1.6 R/S ratio

The R/S ratio *of A. violaceofuscus* and *B. licheniformis* inoculated tomato plants was 1.72 and 2.07-fold higher in sterile Alfisol soil when compared to the negative and positive control, respectively (**Table 2**). Similarly, the R/S ratio of dual inoculated tomato plants in unsterile Alfisol soil were respectively 1.67 and 2.30-fold larger than the negative and positive control. The R/S ratio of dual inoculated tomato plants compared to negative and positive control was 1.48 and 1.72-fold and 1.60 and 1.84-fold higher in sterile and unsterile Vertisol soils, respectively.

#### 3.4.2 Effect of KSMs on K uptake

All the factors and their interactions positively influenced the shoot and root K content of tomato plants except for the two-way interactions soil type × soil condition and mica amendment × soil condition for root K (**Table 1**).

##### 3.4.2.1 Shoot K content

**Figure 4:**
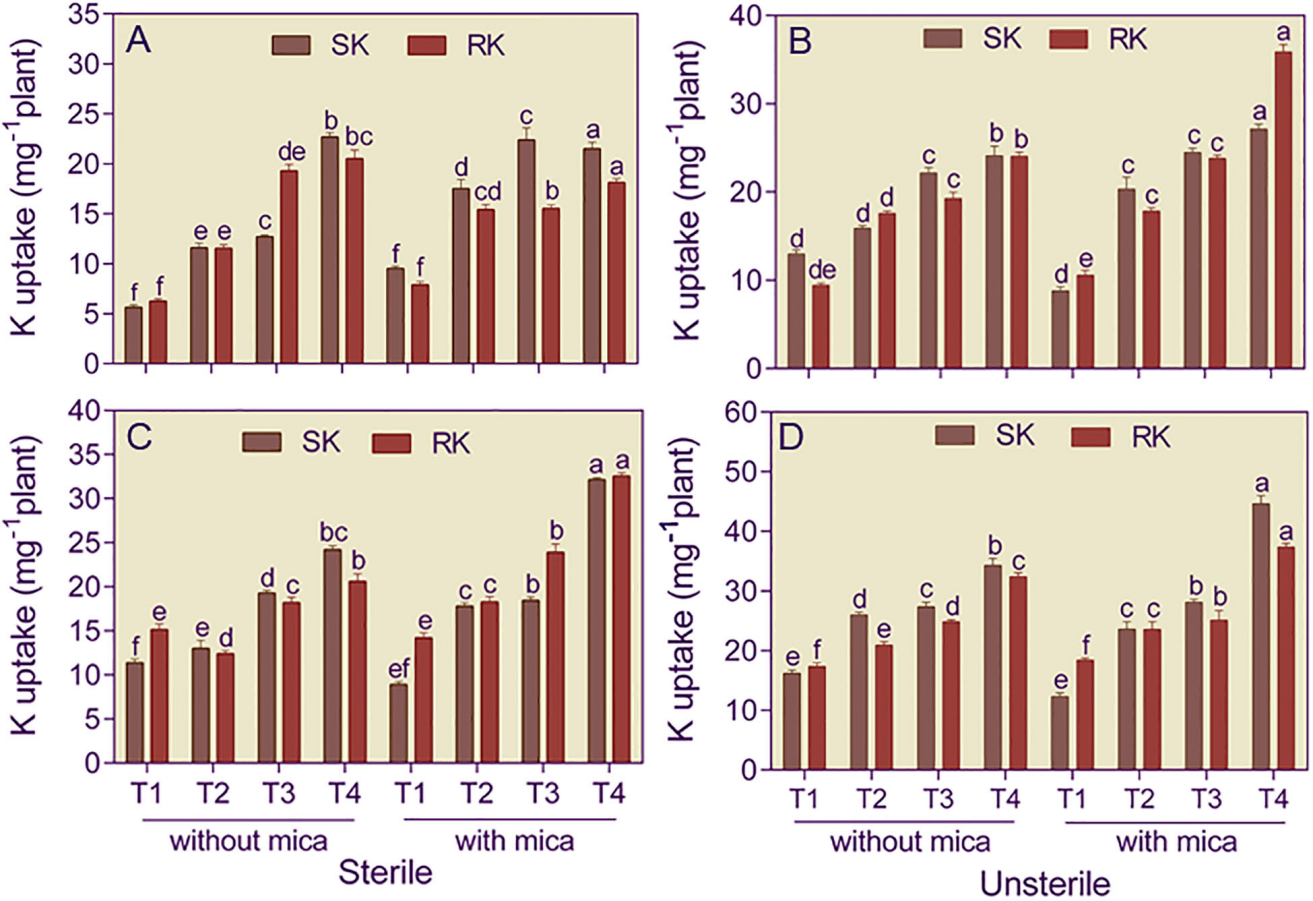
Shoot (SK) and root (RK) potassium content of tomato plants grown in sterile and unsterile Alfisol **(A, B)** and Vertisol **(C, D)** soils amended or unamended with mica and inoculated or uninoculated with potassium solubilizing microorganisms (KSMs). T1, control (negative and positive); T2, *Bacillus licheniformis* inoculation; T3, *Aspergillus violaceofuscus* inoculation; T4, *A*. *violaceofuscus* + *B. licheniformis* inoculation. Bars bearing the same alphabet for a variable are not significantly (p > 0.05) different according to Duncan’s Multiple Range Test.

Shoots of dual inoculated tomato plants raised in Alfisol soil had 4.03 and 8.69-fold and 1.46 and 3.08-fold higher K content than plants in negative and positive control in sterile and unsterile conditions, respectively (**Figures 4A, B**). In Vertisol soil, shoots of dual inoculated tomato plants contained 2.13 and 3.61-fold more K compared to negative and positive control under sterile soil conditions and 2.12 and 3.62-fold more K than negative and positive under unsterile conditions (**Figures 4C, D**).

##### 3.4.2.2 Root K content

Roots of dual inoculated and *B. licheniformis* inoculated tomato plants raised in sterile Alfisol soil had 3.28 and 2.35-fold more K in their roots than the negative and positive controls, respectively (**Figures 4A, B**). However, in unsterile Alfisoil soil, dual-inoculated tomato plants had 4.39 and 3.39-fold more K content than plants of negative and positive control in sterile and unsterile conditions, respectively. In Vertisol soil, roots of dual inoculated tomato plants contained 1.36 and 2.29-fold more K than negative and positive control under sterile soil conditions. In unsterile Vertisol soil, the root K content of dual-inoculated tomato plants was respectively 1.86 and 2.03-fold higher than the negative and positive control (**Figures 4C, D**).

#### 3.4.3 Effect on %RWC

**Figure 5:**
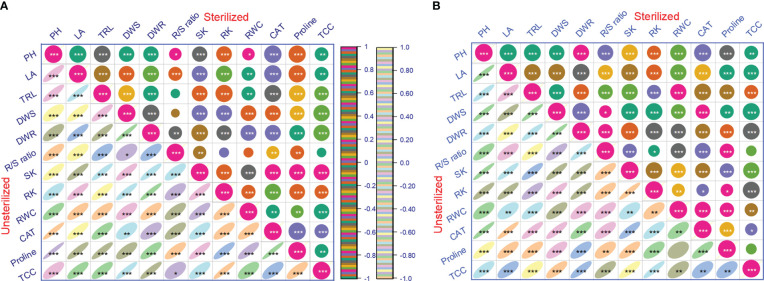
Pearson’s correlation coefficient (r) showing the relationship between growth, potassium content, relative water content, and antioxidant activity variables for tomato plants inoculated with potassium solubilizing microorganisms in the presence or absence of mica in different soil types and conditions (A, Alfisol soil; B, Vertisol soil) under drought (n=24). PH, shoot length; LA, leaf area; TRL, total root length; DWS, shoot dry mass; DWR; root dry mass; SK, shoot potassium content; RK, root potassium content; R/S, root/shoot ratio; RWC, relative water content; CAT, catalase and proline content; TCC, total chlorophyll content. *, **, *** significant at p< 0.05, p< 0.01, p< 0.001 respectively.

Inoculation of tomato plants with *A. violaceofuscus* and *B. licheniformis* with or without mica supplement, alone or in combination, significantly (p< 0.001) increased the %RWC under drought compared to controls. The %RWC was 1.33-fold higher for dual inoculated plants in sterilized Alfisol soil than for plants in the negative control and 1.38-fold higher for plants in the positive control (**Table 3**). The %RWC of co-inoculated plants in unsterilized Alfisol soil was 1.32-fold greater than the negative control and 1.51-fold higher than the positive control. On double inoculation, in sterile Vertisol soil, %RWC was 1.45-fold higher than the negative control and 1.47-fold higher than the positive control. The %RWC of dual inoculated plants in unsterile Vertisol soil was respectively 1.71 and 1.27-fold higher than the negative and positive controls (**Table 3**). All the main factors significantly influenced the %RWC. However, none of the interactions among the factors was significant (**Table 1**). In both the soil types and conditions, the %RWC was significantly and positively correlated with all the investigated variables (**Figures 5A, B**).

#### 3.4.4 CAT activity in tomato plants under drought

All the main factors significantly influenced the CAT activity. Moreover, the two-way interactions mica amendment × microbial inoculation, mica amendment × soil condition, microbial inoculation × soil type, and microbial inoculation × soil condition were significant for CAT activity (**Table 1**). However, the mica amendment × soil condition was not significant for CAT activity. The three-way interactions mica amendment × microbial inoculation × soil type, mica amendment × microbial inoculation × soil condition, and microbial inoculation × soil type × soil condition, were significant for CAT activity, while mica amendment × soil condition × soil type was not significant. The four-way interaction was significant for CAT activity (**Table 1**).

The CAT activity was 2.58 and 2.18-fold greater in tomato plants raised in the sterile Alfisol soil in response to dual inoculation than in the negative and positive controls. In unsterile Alfisol soil, the highest CAT activity (2.57-fold) was observed in sole inoculation of *B. licheniformis* compared with the negative control. Likewise, when compared to the positive control, the CAT activity was 2.11-fold higher in dual-inoculated plants (**Table 3**). In sterile Vertisol soil, the CAT activity was 2.43 and 2.23-fold higher on double inoculation than in the negative and positive control, respectively. In unsterile Vertisol soil, the CAT activity was respectively 1.91 and 2.14-fold higher with double inoculation than in the negative and positive controls (**Table 3**). The CAT activity was significantly and positively correlated with all the plant growth parameters in the different soil types or conditions (**Figures 5A, B**).

**Table 3:** Influence of potassium solubilizing microorganisms (KSMs) on tomato growth under drought condition in different soil type and condition.

Treatment	^†^RWC (%)		Total chlorophyll		Catalase		Proline		
	*ST	*US	ST	US	ST	US	ST		US
Alfisol									
Without mica									
Control	51.26 ± 3.31^d^	58.30 ± 4.89^b^	1.03 ± 0.02^g^	1.29 ± 0.00^e^	13.53 ± 0.31^c^	15.01 ± 0.34^h^	1.01 ± 0.00^e^		1.06 ± 0.02^f^
*Bacillus licheniformis*	53.98 ± 4.33^d^	70.17 ± 2.29^a^	1.11 ± 0.01^f^	1.60 ± 0.02^cd^	31.63 ± 1.51^b^	38.53 ± 0.42^a^	2.82 ± 0.01^bc^		2.91 ± 0.03^d^
*Aspergillus violaceofuscus*	65.73 ± 3.05^bc^	73.38 ± 4.23^a^	1.15 ± 0.00^f^	1.50 ± 0.01^d^	28.91 ± 0.14^c^	29.72 ± 0.29^f^	2.25 ± 0.02^d^		2.52 ± 0.02^e^
*B. licheniformis* + *A. violaceofuscus*	68.32 ± 1.25^bc^	76.85 ± 2.89^a^	1.72 ± 0.00^b^	2.00 ± 0.10^b^	34.96 ± 0.13^a^	35.63 ± 0.30^c^	3.15 ± 0.03^a^		3.20 ± 0.05^b^
With mica									
Control	59.91 ± 4.30^cd^	53.16 ± 0.96^b^	1.25 ± 0.01^e^	1.35 ± 0.01^e^	16.10 ± 0.13^d^	17.53 ± 0.13^g^	0.93 ± 0.02^e^		1.04 ± 0.02^f^
*B. licheniformis*	65.50 ± 5.43^bc^	69.00 ± 3.90^a^	1.54 ± 0.02^c^	1.66 ± 0.02^c^	29.06 ± 0.50^c^	31.77 ± 0.08^e^	2.76 ± 0.15^c^		3.05 ± 0.02^c^
*A. violaceofuscus*	74.70 ± 0.45^ab^	77.90 ± 4.59^a^	1.44 ± 0.01^d^	1.59 ± 0.01^cd^	32.06 ± 0.33^b^	33.15 ± 0.27^d^	2.96 ± 0.03^b^		3.05 ± 0.05^c^
*B. licheniformis* + *A. violaceofuscus*	82.97 ± 3.63^a^	80.05 ± 2.84^a^	2.01 ± 0.03^a^	2.45 ± 0.03^a^	35.15 ± 0.17^a^	36.96 ± 0.41^b^	2.90 ± 0.01^bc^		3.56 ± 0.05^a^
Vertisol									
Without mica									
Control	47.69 ± 4.69^b^	43.54 ± 3.93^d^	1.05 ± 0.01^d^	1.15 ± 0.02^g^	16.15 ± 0.19^f^	21.01 ± 0.42^e^	2.73 ± 0.03^e^		3.13 ± 0.03^d^
*B. licheniformis*	61.92 ± 2.69^a^	56.52 ± 1.38^c^	1.13 ± 0.01^d^	1.24 ± 0.01^f^	35.20 ± 0.22^d^	35.68 ± 0.50^c^	2.92 ± 0.01^d^		3.32 ± 0.03^c^
*A. violaceofuscus*	66.79 ± 2.72^a^	59.17 ± 1.35^bc^	1.29 ± 0.01^c^	1.22 ± 0.01^f^	36.25 ± 0.39^c^	35.20 ± 0.44^c^	2.74 ± 0.05^e^		3.05 ± 0.02d
*B. licheniformis* + *A. violaceofuscus*	69.29 ± 2.27^a^	74.54 ± 8.45^a^	1.61 ± 0.08^b^	1.97 ± 0.01^b^	39.25 ± 0.45^a^	40.15 ± 0.45^a^	3.02 ± 0.01^c^		3.62 ± 0.03^b^
With mica									
Control	48.54 ± 3.71^b^	57.14 ± 4.93^c^	1.36 ± 0.05^c^	1.34 ± 0.01^e^	16.72 ± 0.25^f^	17.91 ± 0.38^f^	1.11 ± 0.02^f^		1.24 ± 0.02^e^
*B. licheniformis*	68.53 ± 3.64^a^	69.05 ± 4.24^abc^	1.58 ± 0.03^b^	1.61 ± 0.01^c^	32.06 ± 0.22^e^	33.49 ± 0.33^d^	3.16 ± 0.04^b^		3.62 ± 0.04^b^
*A. violaceofuscus*	67.17 ± 0.46^a^	78.53 ± 2.80^a^	1.64 ± 0.09^b^	1.48 ± 0.00^d^	34.39 ± 0.53^d^	35.77 ± 0.58^c^	3.04 ± 0.03^c^		3.37 ± 0.05^c^
*B. licheniformis* + *A. violaceofuscus*	71.20 ± 4.03^a^	72.60 ± 2.86^ab^	2.25 ± 0.03^a^	2.56 ± 0.02^a^	37.30 ± 0.25^b^	38.39 ± 0.72^b^	3.33 ± 0.02^a^		4.07 ± 0.07^a^

^†^ %RWC, percentage of relative water content; *Soil condition: ST, sterile; US, unsterile. Means± standard error in a column for a soil type followed by the same superscript(s) is not significantly (p > 0.05) different according to Duncan’s Multiple Range Test.

#### 3.4.5 Proline content in tomato plants under drought

All of the major factors significantly impacted proline content in tomato plants. However, all the two-way, three-way (except microbial inoculation × soil type × soil condition) and four-way interactions among the factors were significant (**Table 1**). Compared to the negative and positive controls, the proline content of tomato plants was 3.11 times and 3.18 times higher in dual inoculated, and *A. violaceofuscus* inoculated plants, respectively, in sterile Alfisol soil. Nevertheless, in unsterile Alfisol soil, the proline content of dual-inoculated tomato plants was 3.0-fold higher than the negative control and 3.41-fold higher than the positive control. Sole inoculation of *A. violaceofuscus* and dual inoculation of the KSMs increased the proline content of tomato plants by 3.18-fold and 3.0-fold, respectively, when compared to the positive and negative controls in unsterile Alfisol soil. Compared to the negative control, dual-inoculated plants had the highest proline content (a three-fold increase) in unsterile Alfisol soil. Similarly, the proline content of co-inoculated plants was 3.41-fold greater than the positive control (**Table 3**).

In sterile Vertisol soil, the dual inoculated tomato plants had 1.10-fold and 2.99-fold higher proline content compared to the negative and positive control, respectively. Similarly, in unsterile Vertisol soil, the dual inoculated tomato plants had 1.16-fold and 3.28-fold higher proline content than the negative and positive control, respectively (**Table 3**). The Proline content, were significantly and positively related to CAT and all plant growth factors and K content in different soil types and conditions (**Figures 5A, B**).

#### 3.4.6 Total chlorophyll content in tomato plants under drought

All the major factors significantly influenced the chlorophyll content of tomato plants (**Table 1**). However, all the two-way, three-way (except microbial inoculation × soil type×soil condition) and four-way correlations were significant for all the studied parameters. All the main factors, except soil type, significantly influenced the total chlorophyll content in tomato leaves. The two-way interaction showed that mica amendment × inoculation, mica amendment × soiltype, soil type × inoculation, soil conidtion × microbial inoculation, and soil condition × soil type were significant for total leaf chlorophyll content. The three-way interactions between microbial inoculation × mica amendment × soil condition and microbial inoculation × soil condition × soil type were significant for all the studied factors. The four-way interaction was significant for leaf total chlorophyll content (**Table 1**). In sterile Alfisol, the total chlorophyll content of dual inoculated plants was respectively 1.67 and 1.61 -fold higher than the negative and positive controls. In unsterile Alfisol soil, the total leaf chlorophyll content of dual inoculated plants was 1.54-fold higher than plants in the negative control, while the total leaf chlorophyll content of *B. licheniformis* inoculated plants was 1.22-fold greater than plants in the positive control. Dual inoculated plants had 1.53 and 1.65-fold higher total leaf chlorophyll content than plants in the negative and positive control, respectively, in sterile Vertisol soil. The total leaf chlorophyll content of co-inoculated plants in unsterile Vertisol soil was 1.72 and 1.90-fold greater than plants in negative and positive controls (**Table 3**). In both the soil type and conditions, the leaf total chlorophyll content was significantly and linearly correlated to all the plant growth parameters, K content, CAT, and proline content (**Figures 5A, B**).

#### 3.4.7 Endorrhizal root colonization in tomato plants under drought

Inoculation of KSMs with or without mica significantly influenced the colonization of native AM and DSE fungi in tomato roots like percentage root length with intracellular linear hyphae (H)/hyphal coils (HC), arbuscules (AR)/arbusculate coils (AC), vesicles (V), DSE fungal hyphae (DSH), moniliform cells (MC) and total root length colonization (RLTC) (**Supplementary Figure S1**). Bioinoculation of KSMs significantly influenced the %RLTC of AM and DSE fungi and root length with different AM and DSE fungal structures in tomato roots (**Table 4**). The AM and DSE fungal structures were absent in tomato roots raised in sterile soils. In Alfisol soil, %RLH, %RLHC, %RLV, and %RLTC were 1.38, 0.62, 0.68, and 1.07-fold higher in dual-inoculated plants, respectively. The %RLAR and %RLMC of *B. licheniformis* inoculated plants were 1.15 and 1.25-fold higher, respectively. Similarly, compared to the negative control, the %RLAC and %RLDSH of plants inoculated with *A. violaceofuscus* alone were 1.33 and 1.19-fold higher, respectively. The %RLV, %RLMC, and %RLTC of dual inoculated plants were respectively 1.12, 1.53, and 1.10-fold higher than the positive control. However, the %RLH and %RLAC of *B. licheniformis* inoculated plants were 1.52 and 0.87-folds higher than the positive control. The %RLHC, %RLAR, and %RLDSH of *A. violaceofuscus* inoculated plants were 1.37, 1.49, and 0.75-fold higher than the positive control (**Table 4**).

Similarly, in the Vertisol soil, the %RLH and %RLTC of dual inoculated plants were 1.75 and 0.99-fold greater than plants in the negative control. However, the %RLV and %RLDSH of plants inoculated with *B. licheniformis* was 0.98 and 1.06-fold higher than the negative control. The %RLHC, %RLAR, %RLAC, and %RLMC of *A. violaceofuscus* inoculated plants were 1.29, 1.16, 1.83, and 2.10-fold higher when compared to the negative control plants. Compared to the positive control, the %RLHC, %RLAC, %RLV, and %RLTC of dual inoculated plants were 0.57, 1.18, 0.58, and 1.04-fold higher. The %RLH, %RLAR, %RLDSH, and %RLMC of *A. violaceofuscus* inoculated plants were 1.91, 1.23, 0.99, and 0.79-fold higher than plants in the positive control (**Table 4**). Mica supplementation significantly affected AM and DSE root colonization by indigenous fungi, as well as %RLHC, %RLV, %RLDSH, and %RLMC. However, KSMs inoculation significantly influenced AM and DSE fungal parameters, except %RLAC and %RLMC. Furthermore, all of the AM and DSE fungal variables, except for %RLMC and %RLTC, were substantially influenced by soil type. The two-way interaction, microbial inoculation×mica amendment, was significant for all AM and DSE fungal variables except %RLAR, %RLV, and %RLMC. For %RLAC and %RLDSH, the interaction soil type × mica amendment was not significant. The interaction, microbial inoculation × soil type, was considerable for all the fungal variables except RLAC and RLDSH. Except for RLAC and RLDSH, the three-way interaction microbial inoculation × mica amendment × soil type was significant for all endophytic fungal variables (**Table 5**).

**Table 4:** Arbuscular mycorrhizal (AM) and dark septate endophytic (DSE) fungal in tomato roots inoculated with potassium solubilizing microorganisms in the presence and absence of mica under drought conditions in different soil types.

Treatment	^†^AM colonization					^††^DSE colonization		
	RLH%	RLHC%	RLAR%	RLAC%	RLV%	RLDSH%	RLMC%	RLTC %
Alfisol								
Without mica								
Control	40.42 ± 0.94^c^	9.04 ± 0.50^a^	6.38 ± 0.93^ab^	5.85 ± 0.51^bc^	9.04 ± 0.50^a^	9.04 ± 0.50^b^	5.88 ± 1.96^a^	88.31 ± 1.91^b^
*Bacillus licheniformis*	46.75 ± 1.47^b^	4.17 ± 0.47^bc^	4.74 ± 1.03^ab^	7.80 ± 0.58^a^	6.04 ± 1.30^b^	10.73 ± 0.60^b^	6.56 ± 0.35^a^	90.42 ± 0.42^ab^
*Aspergillus violaceofuscus*	53.17 ± 1.65^a^	4.73 ± 0.89^bc^	7.37 ± 0.51^a^	2.63 ± 0.52^e^	1.57 ± 0.90^c^	9.48 ± 0.95^b^	7.36 ± 1.36^a^	87.36 ± 0.97^b^
*B. licheniformis* + *A. violaceofuscus*	55.62 ± 2.19^a^	5.64 ± 0.59^bc^	6.71 ± 0.83^ab^	2.52 ± 0.43^e^	6.11 ± 0.75^b^	8.20 ± 0.64^b^	5.51 ± 1.57^a^	94.40 ± 1.26^a^
With mica								
Control	34.44 ± 1.92^d^	4.54 ± 0.62^bc^	4.55 ± 0.67^ab^	7.15 ± 0.69^ab^	5.84 ± 1.13^b^	13.62 ± 1.05^a^	5.83 ± 1.94^a^	83.12 ± 0.61^c^
*B. licheniformis*	46.36 ± 1.53^b^	6.21 ± 0.52^b^	6.79 ± 1.00^ab^	4.52 ± 0.57^cd^	6.23 ± 0.61^b^	10.16 ± 0.94^b^	6.77 ± 0.94^a^	89.28 ± 1.41^b^
*A. violaceofuscus*	52.26 ± 1.23^a^	3.93 ± 0.54^c^	4.48 ± 1.09^ab^	6.19 ± 0.59^abc^	4.50 ± 0.58^b^	7.85 ± 1.07^b^	6.19 ± 1.14^a^	87.66 ± 1.98^b^
*B. licheniformis* + *A. violaceofuscus*	51.16 ± 1.00^ab^	4.74 ± 1.02^bc^	4.19 ± 0.71^b^	3.27 ± 0.39^de^	6.54 ± 0.79^ab^	8.48 ± 0.91^b^	8.91 ± 0.29^a^	91.05 ± 1.30^ab^
Vertisol								
Without mica								
Control	29.40 ± 0.40^d^	11.72 ± 0.81^b^	7.16 ± 0.46^a^	7.18 ± 1.23^bc^	10.46 ± 0.61^a^	11.16 ± 0.91^a^	3.93 ± 0.12^c^	90.19 ± 1.09^bc^
*B. licheniformis*	23.73 ± 1.77^e^	15.07 ± 0.25^a^	8.30 ± 1.09^a^	13.10 ± 1.11^a^	5.01 ± 0.46^bc^	11.27 ± 0.44^a^	8.24 ± 0.82^ab^	87.96 ± 0.98^c^
*A. violaceofuscus*	34.86 ± 1.19^c^	11.09 ± 1.19^b^	3.68 ± 0.68^b^	6.67 ± 1.22^bc^	10.25 ± 1.13^a^	11.83 ± 0.49^a^	3.71 ± 0.75^c^	87.32 ± 1.81^cd^
*B. licheniformis* + *A. violaceofuscus*	51.35 ± 2.29^a^	6.00 ± 1.14^cd^	2.70 ± 0.74^b^	7.31 ± 1.25^bc^	4.95 ± 1.03^bc^	10.01 ± 1.15^a^	4.66 ± 0.62^c^	89.34 ± 0.58^bc^
With mica								
Control	21.52 ± 1.13^e^	13.32 ± 1.20^ab^	6.96 ± 0.57^a^	7.55 ± 0.89^bc^	9.45 ± 0.85^a^	13.32 ± 1.20^a^	10.82 ± 0.95^a^	90.49 ± 1.17^bc^
*B. licheniformis*	41.04 ± 1.01^b^	3.84 ± 0.71^d^	8.54 ± 0.82^a^	3.77 ± 1.97^c^	2.40 ± 1.41^c^	13.23 ± 1.08^a^	8.58 ± 0.96^ab^	84.47 ± 0.90^d^
*A. violaceofuscus*	39.82 ± 1.64^b^	7.18 ± 1.13^c^	7.12 ± 0.92^a^	8.87 ± 1.60^ab^	4.79 ± 0.67^bc^	10.69 ± 0.87^a^	6.60 ± 1.66^bc^	91.64 ± 0.73^ab^
*B. licheniformis* + *A. violaceofuscus*	40.27 ± 0.61^b^	7.53 ± 1.08^c^	7.95 ± 0.26^a^	8.91 ± 1.34^ab^	5.48 ± 0.50^b^	11.51 ± 1.29^a^	5.44 ± 1.29^bc^	94.05 ± 0.74^a^

^†^RLH, root length hyphae; RLHC, root length hyphal coil; RLAR, root length arbuscules; RLAC, root length arbusculate coil; RLV, root length vesicles. ^††^RLDSH, root length dark septate hyphae; RLMC, root length moniliform cells; RLTC, root length total colonization. Mean± standard error in a column for a soil type followed by the same superscript(s) is not significantly (p > 0.05) different according to Duncan’s Multiple Range Test.

**Table 5:** Results of the MULTI-ANOVA analysis for the influence of potassium solubilizing microorganisms inoculation and mica amendment on arbuscular mycorrhizal (AM) and dark septate endophyte (DSE) fungal colonization of tomato roots under drought conditions in different soil types.

^†^Source	df	^††^AM and DSE fungal colonization							
		RLH	RLHC	RLAR	RLAC	RLV	RLDSH	RLMC	RLTC
M	1,47	2.073ns	22.966***	1.219ns	0.387ns	5.494*	3.777*	7.949**	0.534ns
I	3,47	113.451***	14.127***	3.537*	2.039ns	15.514***	5.460**	1.439ns	11.252***
ST	1,47	279.917***	94.091***	4.989*	26.568***	3.885*	17.692***	.047ns	0.642ns
M * I	3,47	27.799***	5.841**	1.821ns	12.784***	1.533ns	4.497*	1.404ns	3.730*
M * ST	1,47	6.594*	5.399*	18.672***	2.712ns	6.442*	.242ns	3.273*	9.933**
I * ST	3,47	5.481**	4.462*	3.436*	2.602ns	13.286***	.691ns	3.015*	8.843***
M * I * ST	3,47	13.094***	24.916***	7.449***	1.676ns	6.860***	1.301ns	3.207*	3.368*

^†^M, mica; I, inoculation; ST, soil type.

^††^RLH, root length hyphae; RLHC, root length hyphal coil; RLAR, root length arbuscules; RLAC, root length arbusculate coil; RLV, root length vesicles; RLDSH, root length dark septate hyphae; RLMC, root length moniliform cells; RLTC, root length total colonization.

*, **, *** significant at p< 0.05, p< 0.01, p< 0.001 respectively; ns, not significant.

## 4 Discussion

Microorganisms have developed a variety of mechanisms to tolerate different adverse environmental factors. In this study, *A. violaceofuscus* and *B. licheniformis* tolerated moisture stress and rapidly grew when exposed to different concentrations of PEG of different molecular weights. Hence, examining these KSMs’ capacity to thrive under osmotically stressful circumstances was critical. Similarly, Ghosh et al. (2019) also reported that *Pseudomonas aeruginosa*, *Bacillus tequilensis*, and *B. endophyticus* showed increased growth under PEG-induced osmotic stress. Likewise, Turco et al. (2005) evaluated the *in vitro* mycelial growth of *Phytophthora quercina*, *P. cambivora*, *P. citricola*, and *P. cinnamomi* and showed increased growth on basal media supplemented with PEG with molecular weights of 3350 and 6000. The fresh and dry fungal mass of *A. violaceofuscus* under PEG-induced osmotic stress conditions was considerably higher in the present study. This study shows that KSMs originating from saxicolous habitats can effectively tolerate drought conditions. Microorganisms adopt different physiological mechanisms like the production of exopolysaccharides, heat shock proteins, increased CAT activity, and solutes like glycine betaine, proline, and trehalose that improve enzyme thermotolerance and deter protein denaturation under moisture stress (Bérard et al., 2015). Moreover, the enhanced resource reallocation and reuse also help microorganisms to tolerate drought conditions (Ngumbi and Kloepper, 2016).

In this study, inoculation of drought-adaptive KSMs *A. violaceofuscus* and *B. licheniformis* supplemented with mica greatly influenced plant growth and K uptake compared to uninoculated tomato plants in different soils under drought conditions. The promotion of tomato growth by *A. violaceofuscus* is similar to a recent study in which soybean and sunflower plants inoculated with *Aspergillus favus* and *A. violaceofuscus* showed increased growth under heat stress (Ismail et al., 2019; Ismail et al., 2020). The current findings are consistent with those reported in different studies using different PGPMs and plant species (Waqas et al., 2012; Mishra et al., 2020). In the current study, the inoculation of KSMs and mica supplementation resulted in a greater total root length than uninoculated plants. The improvement in root characteristics can be due to the release of cellular osmotic stress caused by drought by the conditions provided (Mishra et al., 2020). Moreover, the increased K made available by the KSMs and mica amendment could have also contributed to enhanced root growth as K can improve the development and growth of roots (Sustr et al., 2019). Further, PGPMs also have the metabolic machinery to produce and exude plant growth hormones that can promote root growth in crop plants (Shi et al., 2017). The findings of the current study agree with the results of these studies.

The individual or combined inoculation with *A. violaceofuscus* and *B. licheniformis*, along with the mica supplement, ameliorated the effect of drought on the biomass and the R/S ratio of tomato plants. These observations are very similar to those observed by González-Teuber et al. (2018), who reported that inoculation of *Penicillium minio-luteum* in *Chenopodium quinoa* plant remarkably improved the shoot biomass and R/S ratio under drought conditions. The KSMs can have beneficial effects on plant growth directly and indirectly during water-limiting conditions (Kour et al., 2019; Muthuraja and Muthukumar, 2021a; Muthuraja and Muthukumar, 2021b). The drought tolerance of tomato plants could be attributed to the influence of KSMs on improved nutrient availability through organic acid production. Organic acids produced by PGPMs are shown to provide plants with available nutrients under drought stress (Basu et al., 2016). Besides, KSMs can also affect the production of functional biochemicals and their functions in addition to modifications in antioxidant activities, thus improving plant growth under drought conditions (Racić et al., 2018; Gowtham et al., 2020).

Drought stress affects the availability and transport of plant essential nutrients since they are transported to the roots through water in the soil (Ge et al., 2012; Begum et al., 2021). Microorganisms have an important role in making available the nutrients in the soil and their acquisition by roots, thereby indirectly improving plant growth (Begum et al., 2021). This study revealed that tomato plants inoculated with KSMs in mica-amended soils exhibited higher K levels under drought stress. Furthermore, KSMs may enhance the activities of antioxidant enzymes by improving the K content in tomato plants, which is involved in maintaining turgor pressure and reducing oxidative stress (Hemavathi et al., 2011). The findings reported in the current work align with those documented by Vázquez-de-Aldana et al. (2013), who observed a higher level of K in *Festuca rubra* inoculated with the endophytic fungus *Epichloe festucae* under drought.

Previous studies have shown that several environmental factors, including drought stress, can affect the chlorophyll content in plant leaves, leading to a reduction in photosynthesis (Tränkner et al., 2018; Liu et al., 2019; Khayatnezhad and Gholamin, 2021). Drought stress can greatly reduce the photosynthetic efficiency through modifications in leaf area, affecting chlorophyll development, increasing lipid peroxidation, and enhancing chlorophyll degradation (Khayatnezhad and Gholamin, 2021). Hence, there is a presumption that plants possessing an ability to retain higher chlorophyll concentration under drought stress can be more tolerant to moisture stress and maintain higher growth and biomass production. Our results show that combined inoculation of KSMs along with mica application enhanced total chlorophyll content and %RWC of the tomato plants compared to plants in control. Previously, Gowtham et al. (2020) reported a higher %RWC and chlorophyll content in tomato plants inoculated with *Bacillus subtilis* than uninoculated plants under drought stress. Alwhibi et al. (2017) also reported an improved synthesis of chlorophyll in drought-stressed tomato plants on inoculation with *Trichoderma harzianum*, which confirms the results of the present study.

Furthermore, Li et al. (2018b) demonstrated a strong relationship between plant growth and total chlorophyll concentration. This is also evidenced in the present study, where a significant relationship existed between total chlorophyll content in tomato leaves and plant biomass under drought in both soil types. Therefore, the present study hypothesizes that enhanced chloroplast synthesis through increased %RWC and nutrient availability improved the chlorophyll content of drought-stressed tomato plants inoculated with KSMs containing mica fertilizers.

Previously Feng et al. (2019) suggested that KSMs augment the activity of plant antioxidant enzymes under various stress conditions. In this study, CAT activity and proline content were higher in tomato plants inoculated with KSMs under mica supplementation. This is consistent with previous studies involving maize and *Azospirillum brasilense* (Fukami et al., 2018), perennial ryegrass and *Aspergillus aculeatus* (Li et al., 2021), and *Ocimum basilicum* and *Bacillus lentus, A. brasilense*, and *Pseudomonas* sp (Heidari and Golpayegani, 2012).

Generally, KSMs reduced the negative effects of drought stress on tomato plants by increasing proline levels (Shintu and Jayaram, 2015). Gowtham et al. (2020) reported that *B. subtilis* inoculated tomato plants showed promising drought stress tolerance through increased proline secretion, which corroborated our findings. Proline is a key component in maintaining cell membranes and protein structures (Kavi Kishor et al., 2015). Moreover, proline improves the production and storage of energy by inducing nitrogen metabolic developments under stress conditions by replacing water, thus providing stability to the main structures (Qu et al., 2018; Meena et al., 2019). Proline content in plants increases under stress conditions to maintain osmotic potential-inducing tolerance, as evidenced by its correlation with %RWC.

In this study, treatment of KSMs, either with or without mica enrichment, influenced root colonization of tomato plants by native AM and DSE fungi. Even though the relevance of AM fungi in assisting plant development during drought is well recognized, DSE fungi can also assist the host plant during drought. Endorrhizal fungal colonization in tomato roots was reduced by soil moisture stress. This agrees with a previous study, where drought stress was shown to reduce the mycorrhization of plant roots (Chen et al., 2020). Lower colonization rates are most likely due to limited carbon supply from drought-stressed host plants and drought-induced suppression of fungal spore germination and hyphal development in the rhizosphere soil (Amiri et al., 2015). In addition, organic matter is known to impact the function of soil microorganisms such as PGPMs and AM fungi, allowing them to deliver plant nutrients required for healthy growth (Jacoby et al., 2017). Our findings are in accordance with those of Elsharkawy et al. (2012), who investigated the influence of *Fusarium equiseti* on *Funneliformis mosseae* colonization in cucumber plants. Li et al. (2018a) also reported that some crop plants benefit from the fungi under drought stress conditions, which is evidenced by increased root colonization levels of DSE fungi during drought stress. The increased colonization levels of endorrhizal fungi in response to *A. violaceofuscus* and *B. licheniformis* inoculation clearly suggests that these microorganisms can help improve the formation and functioning of endorrhizal symbiosis of plants under drought stress.

## 5 Conclusion

In this study, the KSMs *A. violaceofuscus* and *B. licheniformis* tolerated different levels of PEG-induced moisture stress through increased CAT activity and proline production. Moreover, these KSMs imparted drought tolerance in tomato plants when inoculated individually or dually with or without mica amendment by improving root elongation and total chlorophyll content under water-limiting conditions. The findings of this research further indicate that under drought conditions, amendment of mica, in addition to dual inoculation, considerably increased tomato plant growth and K absorption in both the Alfisol and Vertisol soils. Furthermore, co-inoculation with KSMs enhanced the %RWC of the tomato plants, CAT activity, and proline content. In addition, dual inoculation of A*. violaceofuscus* and *B. licheniformis* had a stimulatory effect on the endorrhizal symbiosis. Nevertheless, additional field research is required to confirm if the plant improvement potential of these KSMs under controlled greenhouse conditions is translated under varied agro-climatic conditions. Our findings suggest that these KSMs originating from the saxicolous habitats possess the potential to be developed as bio-inoculants for improving crop growth in drought-stressed environments.

## Data availability statement

The original contributions presented in the study are included in the article/**Supplementary Material**. Further inquiries can be directed to the corresponding author.

## Author contributions

RM: Conceptualization, methodology, investigation, formal analysis, data curation, writing- original draft. TM: Conceptualization, supervision, validation, writing- review & editing. CN: Conceptualization, supervision, validation, writing- review & editing. All authors contributed to the article and approved the submitted version.
