# Supplementary material for: Drought tolerance of Aspergillus violaceofuscus and Bacillus licheniformis and their influence on tomato growth and potassium uptake in mica amended tropical soils under water-limiting conditions
Source: Front Plant Sci. 2023 Mar 1;14:1114288. doi: 10.3389/fpls.2023.1114288 (PMC10014471; doi:10.3389/fpls.2023.1114288)

**Supplementary Information for**

**Drought tolerance of *Aspergillus violaceofuscus* and *Bacillus licheniformis* and their influence on tomato growth and potassium uptake in mica amended tropical soils under water limiting conditions**

**Authors:**

Raji Muthuraja ^1,2^ Thangavelu Muthukumar ^2^ and Chittamart Natthapol ^1,*^

^1^*Department of soil science, Faculty of Agriculture, Kasetsart University, Bangkok-10900, Thailand.*

*^2^Root and Soil Biology Laboratory, Department of Botany, Bharathiar University, Coimbatore - 641046, Tamil Nadu, India.*

*Corresponding author. Natthapol Chittamart (E-mail address: fagrnpc@ku.ac.th)

**Table of Supplementary Figures**

[**Supplementary Figure 1 |**](#_Toc100043436) Arbuscular mycorrhizal (AM) (**A–N, Q**) and dark septate endophyte (DSE) (**O–P**) fungal colonization in tomato roots inoculated with potassium solubilizing bacteria (KSB, *B. licheniformis*) and fungi (KSF, *A. violaceofuscus*) individually or dually in the presence or absence of mica under drought conditions. (**A** and **B**) Appressorium (a) and extraradical hyphae (eh) of AM fungi on the root surface of dual inoculated plants in Alfisol soil; (**C**) Intracellular hyphae (h) of AM fungi bearing oil droplets (black arrow heads) in the roots inoculated with KSF in Alfisol soil; **(D)** Intracellular AM fungal hyphal coil (hc) and arbusculate coil (ac) in roots of dual inoculated plants in Vertisol soil; **(E)** Arbusculate coil (ac) in roots of KSF inoculated plants in the presence of mica in Alfisol soil; **(F)** Hyphal coils (hc) and arbusculate coil (ac) in KSB inoculated roots in Vertisol soil; **(G)** Intracellular linear hyphae (ih) and arbusculate coil (ac) of AM fungi in root of plants raised in KSF inoculated and mica amended Alfisol soil; **(H, I)** Arbusculate coil (ac) in tomato roots inoculated with KSF and amended with mica in Alfisol soil; **(J)** Arbuscule (ar) and arbusculate coil (ac) in roots of KSB and KSF dual inoculated plants in Vertisol; **(K)** Arbuscular trunk (at) and arbuscule (ar) in KSB and KSF coinoculated roots in Vertisol; **(L)** Intracellular vesicle (v) and arbusculate coils (ac) in roots inoculated with KSB in the presence of mica in Alfisol soil; (**M**) Hyphal (hc) and arbusculate coil (ac) in roots inoculated with KSF in the absence of mica in Vertisol soil; (**N**) Intracellular vesicles (v) of AM fungi in roots of KSB and KSF dual inoculated plants in the presence of mica in Alfisol soil; (**O**) Hyphae of DSE fungi with septa (arrows) in roots of KSB and KSF dual inoculated plants in the presence of mica in Alfisol soil; (**P**) Moniliform cells (mo) in roots of KSB and KSF coinoculated plants in the presence of mica in Vertisol soil; (**Q**) Intraradical spore (sp) of AM fungi and aseptate linear hyphae (arrow head) in the roots of KSB and KSF dual inoculated in the presence of mica in Alfisol soil. Scale bars = 50µm.

**Figure S1**


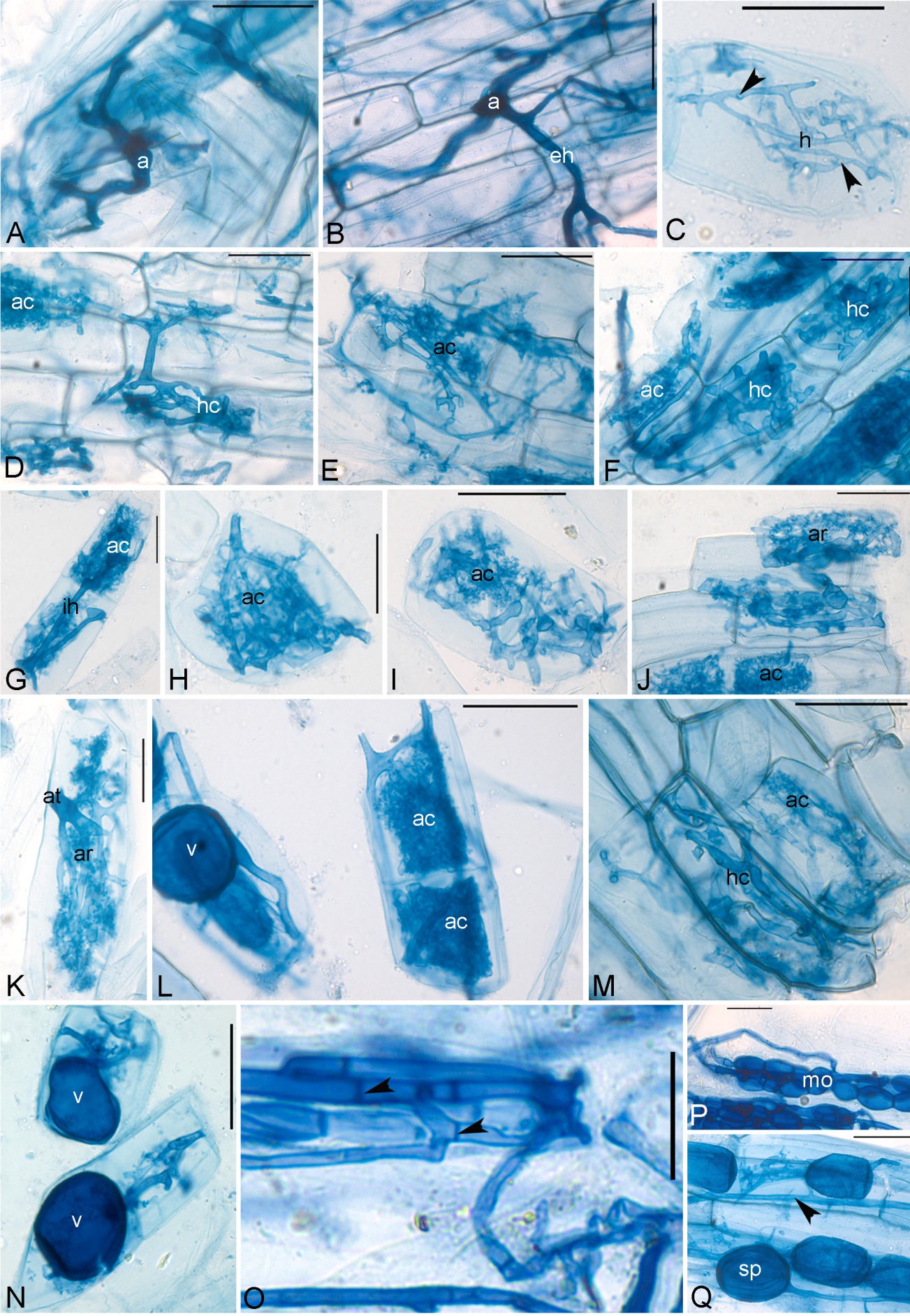

Supplement: Supplementary file 1 [file DataSheet_1.docx]
